# Complete resection of a huge parapharyngeal lesion without neurovascular injuries poses a big challenge

**DOI:** 10.1093/jscr/rjab134

**Published:** 2021-04-22

**Authors:** Ali Hammed, Areeg Alassaf, Salah Hammed, Hala Issa

**Affiliations:** 1 Department of Neurosurgery, Tishreen University Hospital, Lattakia, Syria; 2 Otolaryngology-Head and Neck Surgery, Almouwasat University Hospital, Damascus, Syria; 3 Faculty of Medicine, Aleppo University, Aleppo, Syria; 4 Faculty of Medicine, Tishreen University, Lattakia, Syria

## Abstract

Pleomorphic adenoma is the most common salivary gland neoplasm, accounting for 63% of all parotid gland tumors.

Most tumors originate in the superficial lobe but, more rarely, these tumors may involve the deep lobe of the parotid gland, growing medially and occupying the parapharyngeal space (PPS).

Our case presents a 48-year-old man with an extremely huge recurrent parapharyngeal lesion that bulging in the nasopharynx and the oropharynx and significantly comprised the airways.

Surgery was planned to approach the deeper lobe of parotid gland and para-pharyngeal mass by transparotid, transmandibular swing approach.

Histopathological examination revealed the features suggestive of pleomorphic adenoma. The patient was discharged after 9 days with no facial nerve deficit.

Management of these tumors is more challenging due to the anatomical location of the para-pharyngeal space.

Preoperative definitive diagnosis, with tumor typing, is less important, and incisional biopsy of any PPS mass should definitely be avoided, in order not to run the risk of a significantly higher rate of recurrence.

## INTRODUCTION

Para-pharyngeal space masses account for 0.5% of all head and neck tumors, and the majority is histopathologically benign (76%) [1].

Pleomorphic adenoma is the most common salivary gland neoplasm, accounting for 63% of all parotid gland tumors [2].

Pleomorphic adenomas may occur at any age, but mainly they affect patients in the 3–6 decades [3].

Most tumors originate in the superficial lobe but, more rarely, these tumors may involve the deep lobe of the parotid gland, growing medially and occupying the para-pharyngeal space. Deep lobe parotid pleomorphic adenomas are rare tumors that present a diagnostic and therapeutic challenge. Approximately 10–12% of pleomorphic adenomas of the parotid are thought to arise from the deep lobe of the parotid [4].

Surgical excision is curative; however, as the tumor is poorly encapsulated (despite imaging suggesting otherwise) there is a significant rate of recurrence in the tumor bed. Exact rates of recurrence vary widely depending on series and surgical technique (1–50%) [5].

## CASE PRESENTATION

A 48-year-old male presented with gradually progressive painless swelling of the left upper neck and preauricular region, dysphagia, change in the quality of voice and frequent disturbed sleep during night,

A total of 4 years after undergoing transoral tumor excision for pleomorphic adenoma. On intraoral examination, there was a smooth firm bulge of the soft palate and left lateral pharyngeal wall, occluded the oropharynx. Posterior nasal examination showed the extension of the swelling into the nasopharynx.

**Figure 1:**
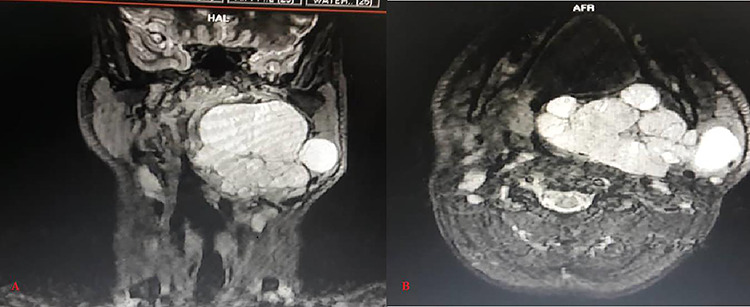
Magnetic resonance STIR WI imaging (**A**: Coronal, **B**: Axial) shows large, lobulated, well defined and hyper intense soft tissue lesion in the left para-pharyngeal space with the lesion extending from the skull base to the hyoid bone, significantly compromising the airways

Magnetic resonance imaging showed large lobulated well-defined homogenously hypointense lesion on T1 WI and hyper intense lesion on T2 and STIR WI in the left para-pharyngeal space extending from the skull base to the hyoid bone. Medially, it was bulging in the nasopharynx and the oropharynx, significantly compromising the airways (Fig. 1).

The deep lobe of the left side parotid gland was not separately identified from the lesion, and it was abutting the neurovascular bundle of the parotid gland from the inner aspect.

**Figure 2:**
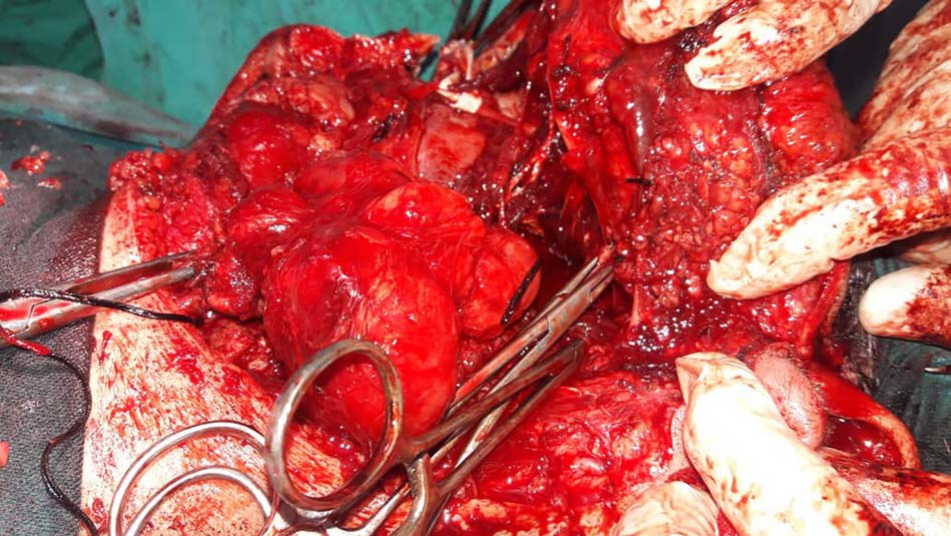
Intraoperative image shows that facial nerve branches around the tumor were dissected out past the mass, so that the tumor could be peeled away from the facial nerve.

**Figure 3:**
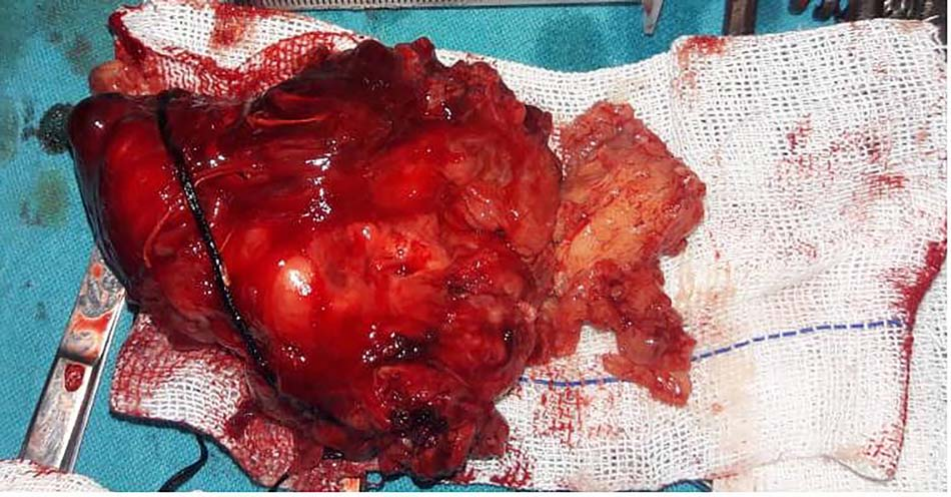
Gross feature of the mass Showing excised specimen of 16 × 10 × 4.5 cm in size

After obtaining the patient’s informed consent, surgery was planned to approach the deeper lobe of parotid gland and para-pharyngeal mass by transparotid, transmandibular swing approach in order to achieve radical excision of the mass. Superficial parotidectomy was done to preserve the facial nerve. After exposure of the parasymphysis, body and ramus of the mandible, mandibular swing access osteotomy was planned. The osteotomy cuts were planned by preserving the inferior alveolar neurovascular bundle, and the media surface of the mandible was exposed to visualize parapharyngeal structures. The facial nerve branches around the tumor were dissected out past the mass, so that the tumor could be peeled away from the facial nerve (Fig. 2). It was completely excised in toto. The measurements of the tumor were 16 × 10 × 4.5 cm in size (Fig. 3). Histopathological examination revealed the features suggestive of pleomorphic adenoma. The patient was discharged after 9 days with no facial nerve deficit.

## DISCUSSION

Parapharyngeal space (PPS) resembles an inverted triangular pyramid with concave faces. Para-pharyngeal space is one of potential facial planes for neoplasms and infections and represents <1% of all head and neck tumors.

PPS tumors are rare, accounting for 0.5% of all head and neck tumors.

Occurrence of the pleomorphic adenoma in the para-pharyngeal space is a rarity [3].

Benign tumors account for ~80%, whereas the remainings are malignant tumors [2]. In the prestyloid space, salivary gland neoplasms (especially parotid gland pleomorphic adenomas) are the most common, whereas neurogenic tumors (e.g. schwannomas and neurofiromas) are those most commonly affecting the poststyloid. Pleomorphic adenoma can develop *de novo* or may arise from deep lobe of the parotid and extends through the stylomandibular tunnel into the PPS [6]. Other less common neoplasms include vascular tumors (paragangliomas), chordomas, lypomas, lymphomas, chemodectomas, rhabdomyomas, chondrosarcomas, desmoid tumors, ameloblastomas, amyloidtumors, ectomesenchymomas, firosarcomas and plasmocytomas [6].

Pleomorphic adenoma in the PPS can develop *de novo* or may arise from deep lobe of the parotid and extend through the stylomandibular tunnel into the PPS [7].

Though these tumors are apparently well encapsulated, resection of the tumor with an adequate margin of grossly normal surrounding tissue where possible is necessary to prevent local recurrence, as these tumors are known to have microscopic pseudopod-like extension into the surrounding tissue due to ‘dehiscences’ in the false capsule [7].

Management of these tumors is more challenging due to the anatomical location of the para-pharyngeal space [8].

To minimize this occurrence, no open surgical biopsy should be performed. Rather, a partial (superficial) or total parotidectomy ensures a wide margin. The facial nerve should be spared using this approach; the recurrence rate has reduced dramatically to 1–4% [9, 10].

## CONCLUSION

Para-pharyngeal space pleomorphic adenomas are infrequent but important tumor of the head and neck. The overall recurrence rate was low at 7.9%, and all recurrences occurred within 5 years of the most recent resection.

The surgical approach should provide excellent visibility with wide surgical exposure to secure local neurovascular structures. Preoperative definitive diagnosis, with tumor typing, is less important and incisional biopsy of any PPS mass should definitely be avoided, in order not to run the risk of a significantly higher rate of recurrence.

## ETHICS APPROVAL

Not required.

## CONSENT TO PUBLISH

Informed written consent was provided by every participant.

## CONFLICT OF INTEREST STATEMENT

All authors declared no conflict of interest.

## FUNDING

None.
